# PFKFB4 Deubiquitination by USP10 Enhances Fumarate Metabolism to Orchestrate the KDM1A/Rad51 Axis and Confer Radioresistance in Lung Cancer

**DOI:** 10.1002/advs.76439

**Published:** 2026-07-08

**Authors:** Yunshang Chen, Zilong Wu, Yongqiang Yang, Ruoxin Fang, Huichan Xue, Rui Zhou, Gang Wu, Xiaohua Jie

**Affiliations:** ^1^ Cancer Center Union Hospital Tongji Medical College Huazhong University of Science and Technology Wuhan China; ^2^ Institute of Radiation Oncology Union Hospital Tongji Medical College Huazhong University of Science and Technology Wuhan China; ^3^ Hubei Key Laboratory of Precision Radiation Oncology Wuhan China

**Keywords:** deubiquitination, fumarate, non‐small cell lung cancer, PFKFB4, radioresistance

## Abstract

Aberrant glucose metabolism reprogramming is a key driver of radioresistance, which is a major obstacle in lung cancer treatment. As a central glucose metabolic regulator, the specific function and molecular mechanisms of PFKFB4 in this process remain undefined. This study revealed that PFKFB4 is aberrantly overexpressed in lung cancer and that targeted inhibition of PFKFB4 enhances radiosensitivity both in vitro and in vivo. Mechanistically, PFKFB4 upregulates fumarate by driving the ATP‐dependent urea cycle. Accumulated fumarate inhibits the histone demethylase KDM1A, leading to increased enrichment of H3K4me1 at the Rad51 promoter and the subsequent transcriptional activation of Rad51, thereby promoting radioresistance. Furthermore, a ubiquitin library screen revealed that the deubiquitinase USP10 is an upstream regulator that binds and stabilizes PFKFB4 by deubiquitinating PFKFB4 at residue K431. Consequently, USP10 depletion increases radiosensitivity by disrupting the PFKFB4/fumarate/Rad51 axis. In summary, this study elucidates the mechanism by which PFKFB4 overexpression confers radioresistance in lung cancer, providing a rationale for targeting this protein in the clinical treatment of lung cancer patients.

## Graphical abstract

USP10 binds to and stabilizes PFKFB4, enhancing glycolytic ATP production, which activates the urea cycle and elevates fumarate. This inhibits histone demethylase KDM1A, leading to increased H3K4me1 enrichment at the Rad51 promoter and direct activation of Rad51 transcription, which confers lung cancer radioresistance. The PFKFB4 inhibitor 5MPN targets this fumarate/Rad51 axis, demonstrating radiosensitizing and translational potential.

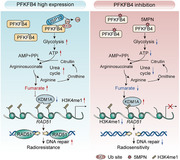

## 1 Introduction

Lung cancer remains the leading cause of cancer‐related mortality worldwide [1]. As a cornerstone treatment for patients with non‐small cell lung cancer (NSCLC), radiotherapy kills tumor cells by inducing DNA double‐strand breaks (DSBs) [2, 3]. However, the efficacy of radiotherapy is often limited by radioresistance. Aberrant glucose metabolism, a recognized characteristic of cancer, supports radioresistance by facilitating energy‐demanding processes such as DSB repair [4, 5]. Furthermore, glucose deprivation sensitizes resistant cells to radiation‐induced apoptosis and growth arrest, underscoring the radioprotective role of glycolytic reprogramming [6]. Consequently, targeting dysregulated glucose metabolism presents a promising avenue for overcoming radioresistance in NSCLC patients.

Phosphofructo‐2‐kinase/fructose‐2,6‐bisphosphatase 4 (PFKFB4), a bifunctional isoenzyme within the PFK2 family, possesses both kinase and phosphatase activities [7]. PFKFB4 regulates fructose‐2,6‐bisphosphate (F2,6P) levels to balance glycolysis and the pentose phosphate pathway (PPP), thereby orchestrating the reprogramming of glucose metabolism in cancer cells [8]. PFKFB4 is upregulated across multiple solid and hematologic malignancies, where it promotes proliferation, metastasis, and apoptosis resistance [9, 10, 11, 12]. As a central node in glucose catabolism, PFKFB4 activates glycolysis, which is essential for DNA repair, and the PPP, which supports nucleotide synthesis and redox homeostasis, indicating that PFKFB4 may be involved in radioresistance [13, 14]. The specific PFKFB4 antagonist 5MPN competitively binds to the fructose‐6‐phosphate binding site, inhibiting the kinase activity of this enzyme [15]. Preclinical studies have shown that 5MPN has high oral bioavailability and exerts antiangiogenic and antiproliferative effects in breast cancer and osteosarcoma models with minimal toxicity toward nontransformed cells [16, 17]. These properties underscore the potential of 5MPN as a tumor‐selective agent with promising clinical translatability.

The hyperactivation of metabolic enzymes may lead to the abnormal accumulation of “oncometabolites”, which can promote the malignant behaviors of tumor cells through epigenetic mechanisms and other pathways [18]. Fumarate, which is primarily generated via the tricarboxylic acid (TCA) cycle and the urea cycle, is a well‐established oncometabolite. Structurally analogous to α‐ketoglutarate (α‐KG), fumarate competitively inhibits α‐KG‐dependent dioxygenases such as histone demethylases (KDMs) and TET DNA demethylases [19]. This epigenetic perturbation stabilizes HIF‐1α, induces epithelial‐mesenchymal transition (EMT), and alters redox homeostasis, collectively increasing tumor cell invasiveness and repopulation capacity [20, 21]. Emerging evidence also implicates fumarate in DSB repair. In yeast, fumarate facilitates the initiation of homologous recombination (HR) to maintain genomic stability [22]. Fumarate accumulation at DSB sites in human cells inhibits KDM2B‐mediated recruitment of the DNA‐PK complex, promoting error‐prone nonhomologous end joining (NHEJ) [23]. Fumarate also attenuates the G2‐M checkpoint, enabling irradiated cells to prematurely re‐enter mitosis, thus increasing their survival [24]. These findings indicate that fumarate contributes to DNA damage repair through epigenetic mechanisms. However, the direct regulatory relationship between fumarate and tumor radiosensitivity has not yet been explored, and whether fumarate influences radioresistance via epigenetic modifications requires further investigation.

In this study, we demonstrated that PFKFB4 is upregulated in lung cancer cells and promotes radioresistance. Genetic silencing or pharmacological inhibition of PFKFB4 with 5MPN increased radiosensitivity both in vitro and in vivo. Mechanistically, PFKFB4 promotes the urea cycle by upregulating ATP production, thus driving the accumulation of fumarate. Subsequent fumarate‐mediated inhibition of the histone demethylase KDM1A increases H3K4me1 enrichment at the Rad51 promoter, activating its transcription and conferring radioresistance. Furthermore, USP10‐mediated deubiquitination of residue K431 stabilizes PFKFB4, thereby establishing the key upstream mechanism that regulates PFKFB4 overexpression. Overall, our findings reveal that the USP10/PFKFB4/fumarate/Rad51 axis drives radioresistance, providing a rationale for targeting PFKFB4 to improve outcomes of lung cancer patients receiving radiotherapy.

## 2 Results

### 2.1 Targeted Inhibition of PFKFB4 Increases the Radiosensitivity of Lung Cancer Cells in Vitro and in Vivo

To evaluate the clinical relevance of PFKFB4, its expression was analyzed in lung cancer using the UALCAN database (https://ualcan.path.uab.edu/). PFKFB4 was significantly upregulated in tumor tissues compared with normal controls (Figure S1A). Western blotting confirmed elevated PFKFB4 protein levels in multiple lung cancer cell lines (A549, H1299, H827, and H460) relative to the normal bronchial epithelial cell line HBE (Figure S1B,C). Immunohistochemistry on lung adenocarcinoma tissue microarrays further showed stronger PFKFB4 staining in tumors than in adjacent normal tissues (Figure S1D,E). Moreover, high PFKFB4 expression correlated with poor patient prognosis (Figure S1F), supporting its oncogenic role in lung cancer.

**FIGURE 1:**
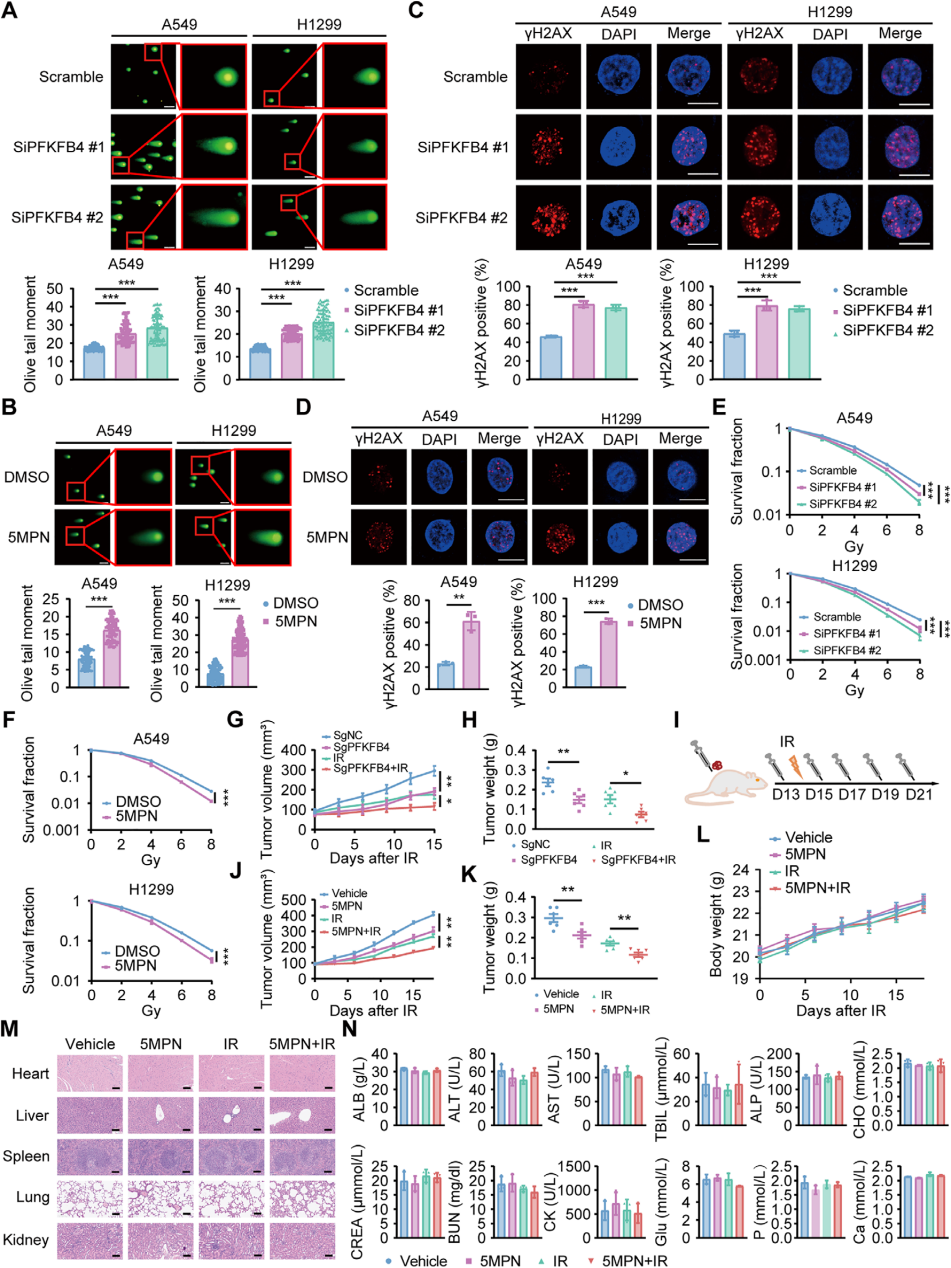
Targeted inhibition of PFKFB4 increases the radiosensitivity of lung cancer cells in vitro and in vivo. (A,B) DNA damage was assessed by the comet assay 4 h after irradiation (6 Gy); tail moments were quantified from the fluorescence microscopy images. n = 100. Scale bar: 50 µm. (C,D) γH2AX foci formation was analyzed 4 h after irradiation (2 Gy). Foci‐positive cells were quantified. n = 3. Scale bar: 10 µm. (E,F) Clonogenic survival assays were performed following exposure to 0, 2, 4, 6, or 8 Gy of irradiation. Colonies were counted on day 14. n = 3. (G) Tumor growth curves based on volume measurements taken every 3 days. n = 6. (H) Final tumor weights per group. n = 6. (I) Schematic of the 5MPN treatment schedule in mice. (J,K) Tumor growth and weight following 5MPN treatment. n = 6. (L) Changes in body weight over time. n = 6. (M) H&E staining images of major organs. Scale bar: 100 µm. (N) Serum biochemical markers across groups. n = 3. Data for in vivo experiments are presented as the means ± SEMs. Abbreviations: ALB: albumin; ALT: alanine aminotransferase; AST: aspartate aminotransferase; TBIL: total bilirubin; ALP: alkaline phosphatase; CHO: cholesterol; CREA: creatinine; BUN: blood urea nitrogen; CK: creatine kinase; Glu: glucose; P: phosphorus; Ca: calcium. Significance is shown in the figure: *p < 0.05; **p < 0.01; ***p < 0.001.

Given the close link between PFKFB4‐involved glucose metabolism and DNA damage repair [13, 14], we investigated whether PFKFB4 influences radiosensitivity. siRNA‑ or small‑molecule inhibitor 5MPN‑mediated PFKFB4 inhibition (Figure S2A; Figure S3) increased comet tail moments and γH2AX foci in irradiated lung cancer cells, indicating enhanced DNA damage (Figure 1A–D). Clonogenic assays revealed reduced survival after irradiation upon PFKFB4 knockdown or pharmacological inhibition, confirming increased radiosensitivity (Figure 1E,F; Figure S4). Notably, 5MPN did not radiosensitize HBE cells, suggesting selective toxicity (Figure S5). We next generated PFKFB4‐knockout lung cancer cells via CRISPR‐Cas9 (Figure S2B) and established subcutaneous xenograft models. Compared with irradiation alone, PFKFB4 knockout combined with irradiation significantly delayed tumor growth (Figure 1G). At the experimental endpoint, the tumors in the combination treatment group had lower weights and exhibited reduced Ki67 staining intensity, supporting the role of PFKFB4 ablation in radiosensitization in vivo (Figure 1H; Figure S6A). Similarly, compared with either treatment alone, 5MPN plus radiotherapy resulted in superior tumor growth suppression (Figure 1I–K; Figure S6B,C). Importantly, the combination regimen did not significantly affect body weight gain (Figure 1L). Hematoxylin and eosin (H&E) staining of major organs and analysis of blood biochemical parameters revealed no notable abnormalities, indicating a favorable safety profile (Figure 1M,N). In summary, PFKFB4 is overexpressed in lung cancer and promotes radioresistance, whereas targeting this enzyme increases radiosensitivity in vitro and in vivo with no overt toxicity.

### 2.2 The Homologous Recombinase Rad51 Is a Critical Downstream Effector of PFKFB4 in Mediating Radioresistance in Lung Cancer

**FIGURE 2:**
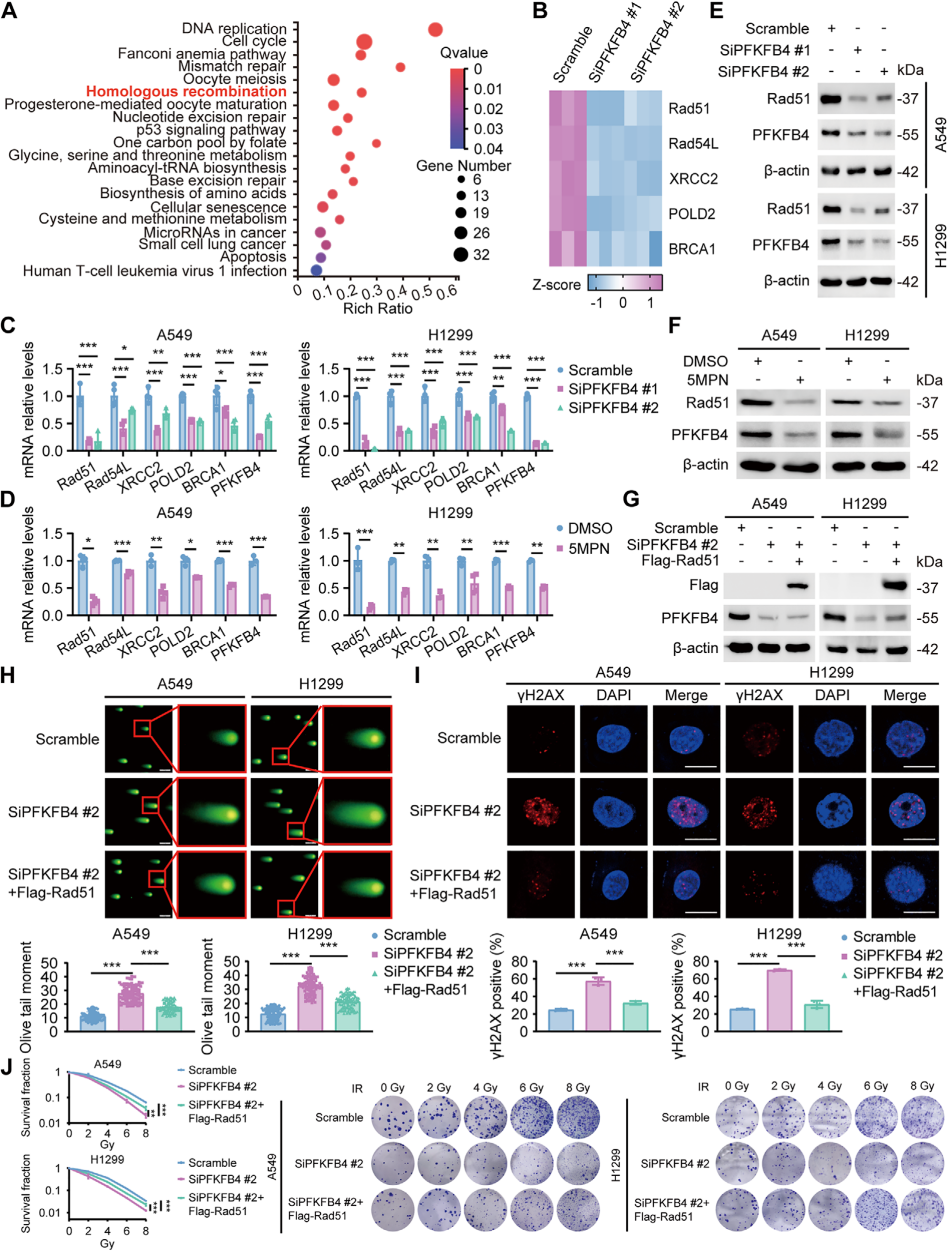
The homologous recombinase Rad51 is a critical downstream effector of PFKFB4 in mediating radioresistance in lung cancer. (A) KEGG pathway enrichment analysis of the DEGs. (B) Heatmap of the expression of genes related to homologous recombination. (C,D) mRNA levels of HR pathway genes after PFKFB4 knockdown (C) and 5MPN treatment (D). n = 4. (E,F) Protein levels of Rad51 after siRNA transfection (E) and 5MPN treatment (F). (G) Validation of siRNA and plasmid transfection efficiency. (H) Comet assay results showing that Rad51 overexpression reverses the DNA damage induced by PFKFB4 silencing. n = 100. Scale bar: 50 µm. (I) The formation of γH2AX foci was reversed by Rad51 overexpression after PFKFB4 knockdown. n = 3. Scale bar: 10 µm. (J) The decrease in clonogenic capacity induced by PFKFB4 silencing was reversed by Rad51 overexpression. Clonogenic survival curves and representative images are shown. n = 3. Significance is shown in the figure: *p < 0.05; **p < 0.01; ***p < 0.001.

To investigate how PFKFB4 promotes radioresistance in lung cancer cells, we performed transcriptome sequencing following PFKFB4 knockdown. Two independent PFKFB4‐targeting siRNAs yielded 849 overlapping differentially expressed genes (DEGs; Q < 0.05, |fold change| ≥ 2) compared with the control (Figure S7). KEGG pathway analysis identified HR, a key DNA double‐strand break repair pathway [25], was significantly enriched among these DEGs (Figure 2A). HR‐related genes (Rad51, Rad54L, XRCC2, POLD2, and BRCA1) were consistently downregulated upon PFKFB4 silencing (Figure 2B). RT‑PCR confirmed that PFKFB4 knockdown or treatment with 5MPN reduced the mRNA levels of HR factors, most notably the core recombinase Rad51 (Figure 2C,D). Western blotting and foci formation assays demonstrated that the Rad51 protein was suppressed following PFKFB4 inhibition (Figure 2E,F; Figure S8A,B). Immunohistochemical (IHC) analysis revealed that 5MPN decreased Rad51 expression in tumors (Figure S8C). Furthermore, rescue experiments revealed that ectopic Rad51 expression abrogated the increased radiosensitivity caused by PFKFB4 deficiency upon irradiation (Figure 2G–J), indicating that Rad51 is a critical downstream effector of PFKFB4. Together, these results establish Rad51 as a key mediator of PFKFB4‐driven radioresistance.

### 2.3 PFKFB4 Activates the Urea Cycle in an ATP‐dependent Manner, Thereby Increasing Fumarate Levels and Ultimately Promoting Rad51 Expression

**FIGURE 3:**
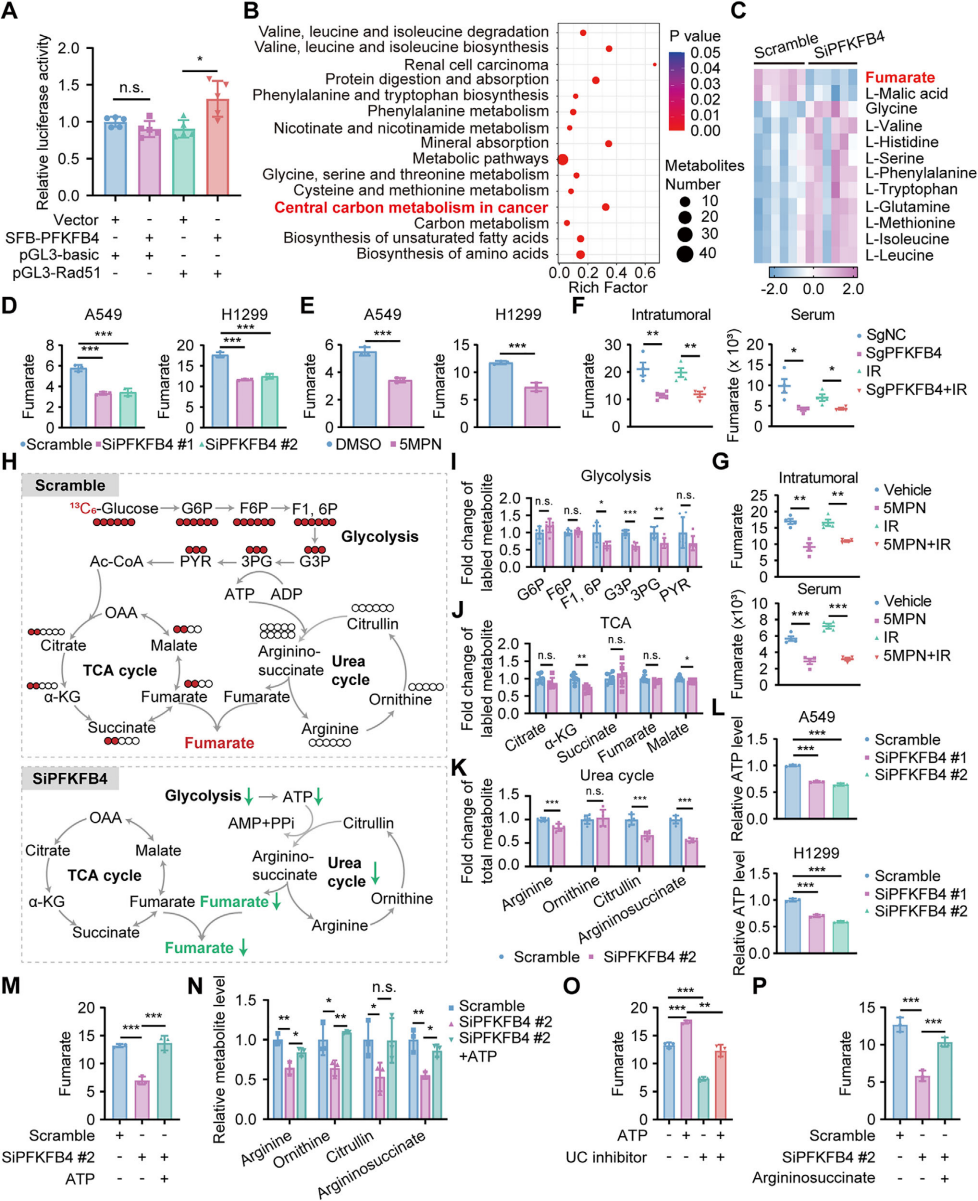
PFKFB4 increases fumarate production by activating the urea cycle through ATP upregulation. (A) Luciferase reporter assay results of Rad51 promoter activity in HEK293T cells transfected with the indicated plasmids. n = 5. (B,C) KEGG enrichment (B) and heatmaps (C) of differentially abundant metabolites between control and PFKFB4‐knockdown H1299 cells. (D,E) Intracellular fumarate levels (nmol/10^6^ cells) following PFKFB4 knockdown (D) and 5MPN treatment (E). n = 3. (F,G) Fumarate levels in xenograft tumors (ng/mg) and mouse serum (nmol/L) measured by LC‐MS. Data are presented as the means ± SEMs. n = 4. (H) Schematic representation of ^1^ ^3^C‐glucose metabolic flux in control and PFKFB4‐knockdown cells. (I,J) Relative levels of [^1^ ^3^C]‐labeled glycolytic (I) and TCA cycle (J) metabolites. n = 6. Abbreviations: G6P: D‐glucose 6‐phosphate; F6P: fructose 6‐phosphate; F1,6P: D‐fructose 1,6‐bisphosphate; G3P: glyceraldehyde 3‐phosphate; 3PG: 3‐phosphoglycerate; PYR: pyruvate. (K) Relative total levels of urea cycle metabolites. n = 6. (L) Intracellular ATP levels after PFKFB4 silencing. n = 3. (M) The intracellular fumarate levels (nmol/10^6^ cells) in each group, with a final ATP concentration of 1 mM. n = 3. (N) Levels of urea cycle metabolites in H1299 cells from each group measured by LC‑MS with a final ATP concentration of 1 mM. n = 3. (O,P) Intracellular fumarate levels (nmol/10^6^ cells) in each group. The CPS1 inhibitor H3B‑120 (1 µM) was applied to inhibit the urea cycle. n = 3. Significance is shown in the figure: n.s., *p* > 0.05; *p < 0.05; **p < 0.01; ***p < 0.001.

PFKFB4 regulates Rad51 expression at both the mRNA and protein levels, suggesting transcriptional control. Indeed, exogenous PFKFB4 overexpression increased Rad51 promoter activity in a dual‐luciferase reporter assay (Figure 3A). Because PFKFB4 is not a canonical transcription factor, we hypothesized that it indirectly regulates Rad51 transcription via metabolic reprogramming, which is consistent with its role as a metabolic enzyme. To identify related metabolites, we performed targeted metabolomics, which revealed distinct alterations upon PFKFB4 silencing (Figure S9). KEGG enrichment analysis of the differentially abundant metabolites revealed that the central carbon metabolism in cancer, which has been strongly linked to tumor radiosensitivity [6], was significantly enriched (Figure 3B). Within this pathway, the level of the metabolite fumarate markedly decreased upon PFKFB4 silencing (Figure 3C). Consistent with this finding, PFKFB4 knockdown or pharmacological inhibition with 5MPN decreased the level of intracellular fumarate in lung cancer cells but not in HBE cells (Figure 3D,E; Figure S10). Similarly, both PFKFB4‐knockout mice and 5MPN‐treated mice presented reduced fumarate levels in both the tumors and serum (Figure 3F,G). These observations collectively confirm the tumor cell‐specific regulation of fumarate by PFKFB4. To elucidate the underlying mechanism, metabolic flux analysis using [^1^ ^3^C_6_]‐glucose was conducted. The findings of this assay revealed that PFKFB4 knockdown did not affect the level of [^1^ ^3^C_2_]‐fumarate in the TCA cycle but did significantly reduce the levels of glycolytic intermediates ([^1^ ^3^C_6_]‐F1,6P, [^1^ ^3^C_3_]‐G3P, and [^1^ ^3^C_3_]‐3PG) and ATP (Figures 3H‐J; Figure 3L). Given that the urea cycle has a high demand for ATP and is a key source of fumarate [26], we next assessed the activity of the urea cycle and found that it was suppressed upon PFKFB4 silencing (Figure 3K). Notably, exogenous ATP supplementation reversed the decrease in fumarate and urea cycle metabolite levels induced by PFKFB4 knockdown (Figure 3M,N). However, the expression of key urea cycle enzymes (CPS1, ASS1, ASL, OTC, and ARG1) [27] was not affected by changes in PFKFB4 expression (Figure S11), suggesting that PFKFB4 regulates the urea cycle and fumarate primarily by modulating energy availability. Furthermore, exogenous ATP significantly increased intracellular fumarate levels, but this effect was abolished upon treatment with a urea cycle inhibitor, confirming the upstream role of the urea cycle in fumarate production (Figure 3O). More importantly, supplementation of PFKFB4‐silenced cells with argininosuccinate—the urea cycle intermediate most prominently regulated by PFKFB4—restored fumarate levels (Figure 3P). Taken together, these results indicate that PFKFB4 primarily activates the urea cycle in an ATP‐dependent manner, thereby promoting fumarate accumulation.

**FIGURE 4:**
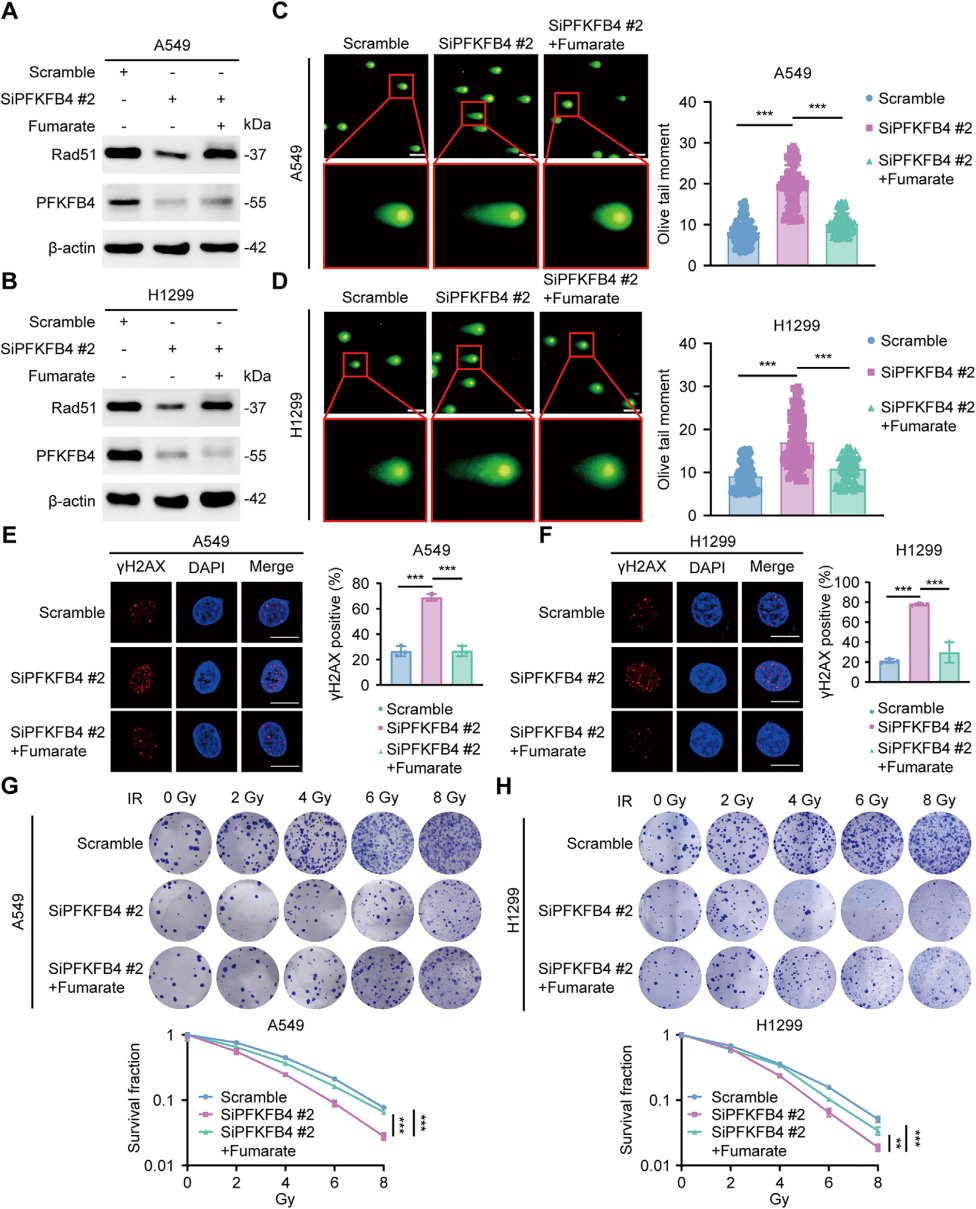
PFKFB4 upregulates Rad51 expression in a fumarate‐dependent manner, thereby inducing radioresistance in lung cancer cells. (A,B) Western blotting showing that fumarate rescues the downregulation of Rad51 induced by PFKFB4 knockdown. (C,D) Representative comet assay images and quantification of comet tail moments across treatment groups. n = 100. Scale bar: 50 µm. (E,F) Representative images of the formation of γH2AX foci and statistical analysis of foci‐positive cells. n = 3. Scale bar: 10 µm. (G,H) Clonogenic survival curves and representative images of cells from each group. n = 3. Significance is shown in the figure: **p < 0.01; ***p < 0.001.

Furthermore, exogenous fumarate rescued the PFKFB4 knockdown‐induced downregulation of Rad51 expression, confirming its fumarate‐dependent regulation (Figure 4A,B). Functionally, PFKFB4 silencing exacerbated radiation‐induced DNA damage (as indicated by increased numbers of comet tails and γH2AX foci) and reduced clonogenic survival, which were partially reversed by fumarate supplementation, directly linking fumarate to PFKFB4‐mediated radioresistance (Figure 4C–H). In summary, PFKFB4 increases glycolytic ATP production to sustain urea cycle activity and fumarate generation, which upregulate Rad51 expression and promote radioresistance in lung cancer.

### 2.4 Fumarate Induces the Transcriptional Activation of Rad51 by Promoting H3K4me1 Enrichment at the Rad51 Promoter Region via the Inhibition of KDM1A

**FIGURE 5:**
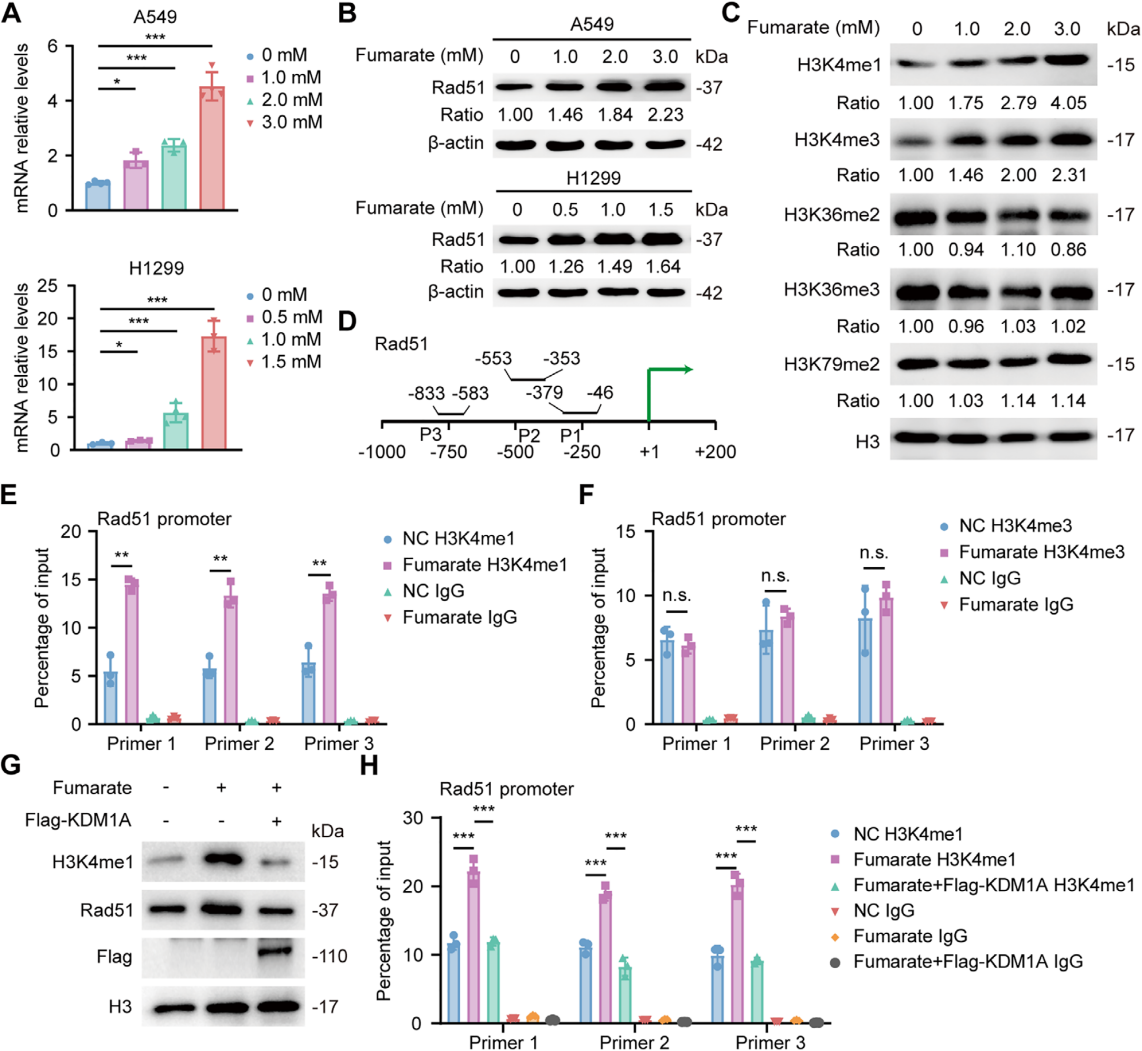
Fumarate induces the transcriptional activation of Rad51 by promoting H3K4me1 enrichment at the Rad51 promoter region via the inhibition of KDM1A. (A,B) Dose‐dependent increases in Rad51 mRNA (A) and protein (B) levels in lung cancer cells after 24 h of monoethyl fumarate treatment. n = 4. (C) Western blotting of histone methylation marks following fumarate exposure. (D) Schematic of ChIP‐PCR primer design for the Rad51 promoter. (E,F) ChIP‐PCR quantification of H3K4me1 (E) and H3K4me3 (F) enrichment at the Rad51 promoter. n = 3. (G) Western blotting showing that fumarate treatment reverses the changes in Rad51 and H3K4me1 levels upon KDM1A overexpression. (H) ChIP‐PCR was performed to determine H3K4me1 enrichment at the Rad51 promoter in each group. n = 3. Significance is shown in the figure: n.s., *p* > 0.05; *p < 0.05; **p < 0.01; ***p < 0.001.

Although fumarate links PFKFB4 expression to Rad51 expression, the mechanism by which Rad51 expression is regulated remains unclear. Exogenous fumarate supplementation increased the Rad51 mRNA and protein levels in a concentration‐dependent manner (Figure 5A,B). As an epigenetic modulator, fumarate competitively inhibits α‐KG‐dependent dioxygenases, including histone demethylases (KDMs), thereby altering histone methylation levels and transcriptional activity [28, 29]. To determine whether fumarate regulates Rad51 via this pathway, we examined previously reported fumarate‐regulated histone marks associated with transcriptional activation (H3K4me1, H3K4me3, H3K36me2, H3K36me3, and H3K79me2) [30, 31]. This analysis revealed that the levels of only H3K4me1 and H3K4me3 increased in lung cancer cells after fumarate treatment (Figure 5C). Subsequent ChIP‐PCR revealed increased H3K4me1 enrichment specifically at the Rad51 promoter, with no change in the enrichment of H3K4me3 (Figure 5D–F). Because KDM1A is the principal H3K4me1 demethylase [32], we next evaluated its role in this process. KDM1A overexpression reversed the fumarate‐induced increases in Rad51 expression and H3K4me1 levels (Figure 5G) and abolished H3K4me1 enrichment at the Rad51 promoter (Figure 5H). However, other reported histone demethylases associated with Rad51 (KDM4A, KDM4C, and KDM5B) [33, 34, 35] were not involved in the fumarate‐mediated regulation of Rad51 (Figure S12). These findings indicate that fumarate triggers Rad51 transcription by inhibiting KDM1A, leading to H3K4me1 accumulation at the Rad51 promoter.

### 2.5 USP10 Stabilizes PFKFB4 Expression Through Direct Binding and Deubiquitination of PFKFB4 Residue Lysine 431

**FIGURE 6:**
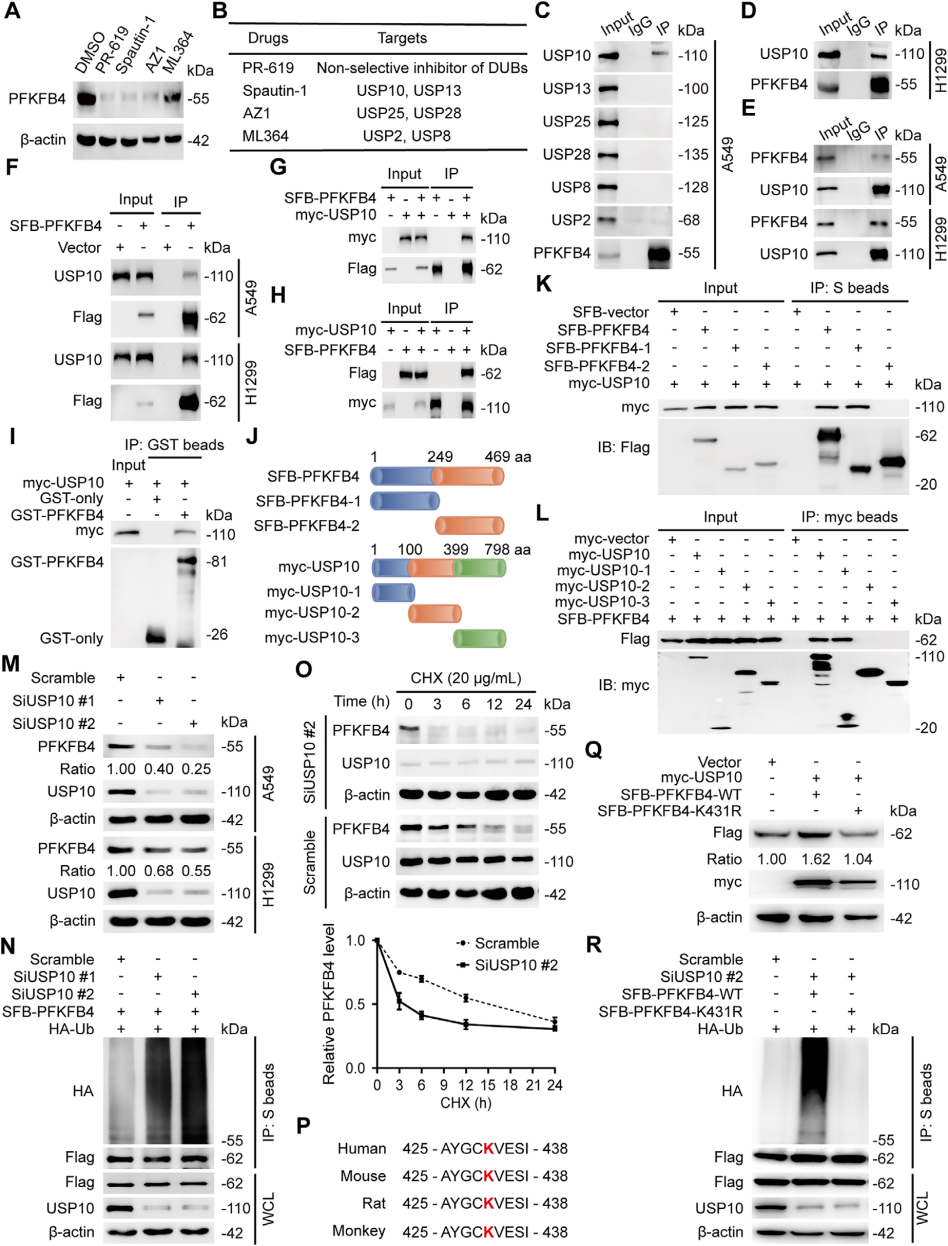
USP10 stabilizes PFKFB4 expression through direct binding and deubiquitination at lysine 431. (A) Screening revealed four DUB inhibitors that decreased PFKFB4 protein levels. (B) List of DUB inhibitors and their targets. (C–E) Endogenous co‐IP of PFKFB4 and USP10 in A549 and H1299 cell lysates. (F) Co‐IP of endogenous USP10 using exogenous SFB‐PFKFB4. (G,H) Exogenous co‐IP in HEK293T cells transfected with tagged PFKFB4 and USP10 plasmids. (I) The results of the GST pull‐down assay confirming the direct interaction between USP10 and PFKFB4. (J) Schematic of the PFKFB4 and USP10 domain truncation. (K,L) Mapping of the interaction domains by co‐IP using the truncated constructs. (M) USP10 knockdown reduces PFKFB4 protein levels. (N) USP10 silencing increases PFKFB4 ubiquitination under MG132 treatment. (O) CHX chase assay results showing the half‐life of the PFKFB4 protein upon USP10 knockdown. (P) Evolutionary conservation of PFKFB4 K431. (Q) USP10 overexpression upregulates the expression of wild‐type (WT) but not K431R‐mutant PFKFB4. (R) USP10 silencing increases the ubiquitination of wild‐type (WT) but not K431R‐mutant PFKFB4.

Although PFKFB4 is known to undergo FBXW7‐mediated ubiquitination and degradation [36], the mechanisms regulating its deubiquitination remain unclear. To identify upstream regulators of PFKFB4 stability, we screened a ubiquitination‐focused compound library comprising 198 small‐molecule inhibitors (Figure S13). This screen revealed four deubiquitinase (DUB) inhibitors that markedly reduced PFKFB4 expression (Figure 6A); the targets of these inhibitors are listed in Figure 6B. Coimmunoprecipitation (co‐IP) validation revealed a specific interaction between PFKFB4 and USP10 (Figure 6C), suggesting that USP10 is a potential regulator. This interaction was confirmed by exogenous and endogenous co‐IP assays across multiple cell lines (Figure 6D–H). Notably, the results of the GST pull‐down experiments revealed a direct interaction between the two proteins (Figure 6I). Using truncation mutants based on structural features [37], we determined that the kinase domain of PFKFB4 (aa 1–249) and the N‐terminal domain of USP10 (aa 1–100) are required for their interaction (Figure 6J–L). These findings establish USP10 as a bona fide interacting partner of PFKFB4. To define the regulatory effect of USP10 on PFKFB4, we silenced USP10 in lung cancer cells, which resulted in reduced PFKFB4 protein levels (Figure 6M). Consistent with this observed reduction, USP10 knockdown promoted PFKFB4 ubiquitination and accelerated its degradation, indicating that USP10 stabilizes PFKFB4 through deubiquitination (Figure 6N,O). To map the USP10‐mediated deubiquitination site on PFKFB4, mass spectrometry was performed, and the highly conserved lysine 431 (K431) residue was identified (Figure 6P; Data file S1). Additional validation experiments demonstrated that mutation of K431 abolished the USP10‐mediated expression and deubiquitination of PFKFB4 (Figure 6Q,R). Together, these results demonstrate that USP10 directly binds to PFKFB4 and promotes its deubiquitination at K431, thereby stabilizing PFKFB4 in a proteasome‐dependent manner.

### 2.6 USP10 Activates the Fumarate/Rad51 Axis by Stabilizing PFKFB4 to Promote Radioresistance in Lung Cancer Cells

**FIGURE 7:**
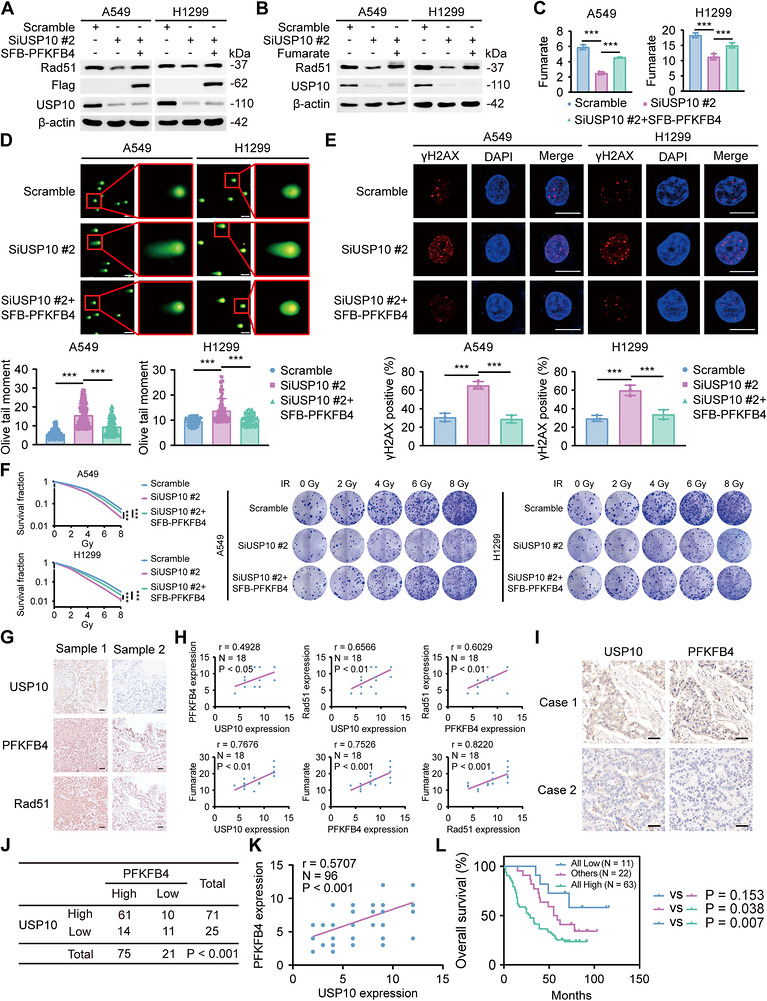
USP10 activates the fumarate/Rad51 axis by stabilizing PFKFB4 to promote lung cancer cell radioresistance. (A) Western blotting showing that PFKFB4 overexpression rescued the downregulation of Rad51 expression caused by USP10 silencing. (B) Western blotting showing that fumarate reverses the decrease in Rad51 expression induced by USP10 silencing. (C) Intracellular fumarate levels (nmol/10^6^ cells) in the indicated groups. n = 3. (D) Neutral comet assay results demonstrating that the increase in DNA damage caused by USP10 knockdown is reversed by PFKFB4 overexpression. n = 100. Scale bar: 50 µm. (E) γH2AX immunofluorescence staining showing that PFKFB4 overexpression reverses the increase in DNA damage induced by USP10 silencing. n = 3. Scale bar: 10 µm. (F) Clonogenic survival assays results showing that radiosensitivity is increased by USP10 knockdown and rescued by PFKFB4 overexpression. n = 3. (G) Representative IHC staining images of USP10, PFKFB4, and Rad51 in mouse subcutaneous tumor tissues. Scale bar: 50 µm. (H) Correlations between fumarate levels and the USP10/PFKFB4/Rad51 axis in mouse subcutaneous tumor tissues. n = 18. (I) Representative IHC staining images of USP10 and PFKFB4 in paired lung adenocarcinoma samples. Scale bar: 50 µm. (J) Lung adenocarcinoma tissue microarray samples were categorized by IHC score for USP10 and PFKFB4. An IHC score of ≥ 6 was defined as high expression. n = 96. (K) A significant positive correlation was detected between USP10 and PFKFB4 expression in lung cancer samples. n = 96. (L) Kaplan‐Meier survival analysis of patients stratified by combined USP10/PFKFB4 expression. Significance is shown in the figure: ***p < 0.001.

To elucidate how USP10 participates in conferring radioresistance in lung cancer cells and determine whether this effect is mediated through PFKFB4, we exogenously overexpressed PFKFB4 in USP10‐deficient lung cancer cells. Western blotting revealed that USP10 knockdown not only decreased PFKFB4 protein levels but also significantly reduced Rad51 expression. Notably, both PFKFB4 overexpression and exogenous fumarate supplementation effectively restored Rad51 expression in USP10‐depleted cells (Figure 7A,B). Furthermore, intracellular fumarate levels decreased upon USP10 silencing, and this reduction was reversed by exogenous PFKFB4 overexpression (Figure 7C). Functionally, USP10 knockdown increased the number of comet tail moments, elevated γH2AX foci formation, and reduced clonogenic survival following irradiation, all of which were reversed by PFKFB4 overexpression (Figure 7D–F). These findings demonstrate that USP10 stabilizes PFKFB4 expression to activate the fumarate/Rad51 axis, thereby promoting radioresistance in lung cancer. To validate the biological significance of the fumarate‐USP10/PFKFB4/Rad51 axis in vivo, we analyzed samples from mice bearing subcutaneous tumors. The results revealed significant pairwise positive correlations between USP10, PFKFB4, and Rad51 expression and positive correlations between intratumoral fumarate levels and that of each of these molecules (Figure 7G,H). To further explore the clinical translational value of the USP10/PFKFB4 axis, we performed IHC staining of paired lung adenocarcinoma tissue microarrays, which revealed a significant positive correlation between USP10 and PFKFB4 expression (r = 0.5707; Figure 7I‐K). Stratified survival analysis revealed that USP10^high^PFKFB4^high^ patients had shorter overall survival than USP10^low^PFKFB4^low^ patients did (median survival: 29 months vs. not reached), indicating that USP10 and PFKFB4 coexpression is a prognostic marker for poor clinical outcomes in patients with lung cancer (Figure 7L). Additionally, pan‐cancer analysis of the GEPIA database demonstrated that high PFKFB4 expression was significantly linked to poor patient prognosis (Figure S14A–D). The positive correlation between USP10 and PFKFB4 expression was also observed across multiple malignancies (Figure S14E–H), reinforcing the notion that the USP10/PFKFB4 axis is widely implicated in cancer biology.

## 3 Discussion

This study provides the first evidence that PFKFB4 induces radioresistance in lung cancer cells. Mechanistically, USP10 binds and stabilizes PFKFB4 via its deubiquitination at K431. The accumulated PFKFB4 upregulates ATP levels, thereby activating the urea cycle and subsequently increasing fumarate production. This elevated fumarate inhibits KDM1A to increase H3K4me1 enrichment at the Rad51 promoter, ultimately enhancing Rad51 transcription and radioresistance. Furthermore, 5MPN specifically affects PFKFB4 function, thereby suppressing the activity of the fumarate/Rad51 axis to achieve tumor radiosensitization. Notably, 5MPN is safe and tolerable in vivo, highlighting its promising clinical translational potential.

Glucose metabolism reprogramming allows tumor cells to rapidly generate ATP and biosynthetic precursors, which are critical for proliferation, progression, and radioresistance [38, 39]. PFKFB4, a key regulator of glycolysis, is involved in diverse biological processes, such as cell cycle control, proliferation, and antiapoptotic signaling [40, 41]. However, its role in tumor radiosensitivity remains poorly defined. Here, we report that PFKFB4 knockdown markedly reduces the clonogenic survival of irradiated lung cancer cells and exacerbates radiation‐induced DNA damage, suggesting that the loss of PFKFB4 increases radiosensitivity. These findings were corroborated in vivo, where compared with radiation alone, PFKFB4 knockout synergized with irradiation to suppress tumor growth more effectively. Transcriptome analysis revealed that PFKFB4 depletion led to significant enrichment of the HR pathway, which is essential for repairing DSBs. PFKFB4 silencing led to the downregulation of Rad51, a core HR factor, identifying Rad51 as a key effector of PFKFB4‐driven radioresistance. Subsequent rescue experiments confirmed this causal link. These data reveal an intrinsic connection between PFKFB4 and DNA damage repair, clarifying the mechanism underlying the PFKFB4‐mediated regulation of radiosensitivity. Moreover, 5MPN, a specific PFKFB4 inhibitor, has been shown to demonstrate antitumor activity and high oral bioavailability in preclinical models [13, 16]. Notably, 5MPN acts synergistically with radiotherapy to exert potent antitumor activity. Importantly, the combination regimen caused no adverse effects on murine growth or vital organ function, indicating a favorable safety profile in vivo. The observed synergy between 5MPN and radiotherapy may thus overcome radioresistance and improve therapeutic outcomes, offering a promising novel strategy for the treatment of advanced lung cancer.

Cellular metabolism and gene expression engage in bidirectional regulation [42]. Gene expression adaptively controls metabolic processes, whereas metabolites and metabolic enzymes reciprocally influence chromatin dynamics and gene expression [43, 44]. This study revealed that PFKFB4 expression controls intracellular fumarate levels, with targeted PFKFB4 inhibition depleting fumarate both in vitro and in vivo. ^13^C‐glucose flux analysis indicated that fumarate reduction following PFKFB4 suppression resulted from urea cycle impairment, rather than TCA cycle dysfunction, due to diminished glycolysis‐driven ATP. Fumarate, in turn, upregulates Rad51 expression and reverses its downregulation following PFKFB4 knockdown, identifying fumarate as the key metabolic mediator between PFKFB4 and Rad51. Mechanistically, fumarate promotes Rad51 transcription via epigenetic remodeling. Exogenous fumarate supplementation increased H3K4me1 enrichment at the Rad51 promoter, an effect that was reversed by KDM1A overexpression, indicating that fumarate‐mediated transcriptional activation involves competitive inhibition of the histone demethylase KDM1A. Consequently, H3K4me1 accumulation at the Rad51 promoter occurs, increasing its transcription. Functionally, fumarate supplementation reversed the radiosensitization phenotypes induced by PFKFB4 silencing, demonstrating that the fumarate/Rad51 axis underlies PFKFB4‐driven radioresistance in lung cancer cells. In summary, PFKFB4 activates the ATP‐dependent urea cycle to elevate fumarate, which functions as an endogenous epigenetic modulator to promote Rad51 expression via promoter chromatin remodeling. This metabolism‐epigenetics cascade illustrates how metabolic reprogramming drives radioresistance in lung cancer cells.

Ubiquitination and deubiquitination serve as critical posttranslational modifications that regulate protein stability and function, often acting as “molecular switches” in the precise control of cellular metabolic processes [45, 46]. In cancer cells, the ubiquitin‐proteasome system frequently remodels metabolism by regulating key enzymes [47, 48, 49]. Here, the upstream deubiquitination mechanism governing the glycolytic enzyme PFKFB4 and its role in metabolic reprogramming and radioresistance were elucidated for the first time. USP10 was identified as a direct PFKFB4 binding partner from a ubiquitination compound library screen, with the interaction interface mapped to the USP10 N‐terminal domain (aa 1–100) and the PFKFB4 kinase domain (aa 1–249). USP10 silencing reduced the abundance of the PFKFB4 protein, increased its ubiquitination, and shortened its half‐life, establishing USP10 as a key deubiquitinase that stabilizes PFKFB4. Mass spectrometry revealed K431 as the primary ubiquitination site on PFKFB4, and its mutation abolished USP10‐mediated stabilization and deubiquitination, directly explaining the observed PFKFB4 overexpression in lung cancer cells. Clinically, USP10 and PFKFB4 levels were strongly positively correlated in lung cancer specimens, and their coexpression predicted poor patient outcomes. Given the established role of the USP10/PFKFB4 axis in radioresistance, this coexpression pattern may serve as a valuable biomarker for predicting the response to radiotherapy and survival outcomes.

In summary, this study identifies PFKFB4 as a pivotal regulator of radioresistance in lung cancer cells. We revealed a pathway through which PFKFB4‐driven metabolic‐epigenetic reprogramming increases DNA repair, contributing to therapeutic failure. Importantly, inhibition of PFKFB4 with 5MPN restored radiosensitivity, suggesting that this strategy is promising for overcoming treatment resistance.

## 4 Experimental Section

### 4.1 Cell Culture and Transfection

The A549 (RRID: CVCL_0023), H1299 (RRID: CVCL_0060), H827(RRID: CVCL_2063), H460 (RRID: CVCL_0459), HBE (CVCL_VP25), and HEK‐293T (RRID: CVCL_0063) cells used in this study were purchased from the American Type Culture Collection (ATCC) and authenticated by short tandem repeat (STR) profiling. All the cell lines were confirmed to be mycoplasma‐free prior to experimentation. siRNA transfection of cells in the logarithmic growth phase at 30%–40% confluence was performed using Lipofectamine RNAiMAX. Gene‐specific siRNAs targeting PFKFB4 and USP10 were synthesized by JTSBIO Co., Ltd., with the following sequences:

Scramble siRNA: 5'‐UUCUCCGAACGUGUCACGU‐3';

siPFKFB4 #1: 5'‐GCAUCGUAUAUUACCUCAUTT‐3';

siPFKFB4 #2: 5'‐GCAAGACCUACAUCUCCAATT‐3';

siUSP10 #1: 5'‐CCCUGAUGGUAUCACUAAAGA‐3'; and

siUSP10 #2: 5'‐CGACAAGCUCUUGGAGAUAAA‐3'.

### 4.2 Antibodies and Reagents

The following antibodies were used in this study: rabbit anti‐PFKFB4 (Abcam, ab137785, RRID: AB_2722775, 1:500); rabbit anti‐Rad51 (Abcam, ab63801, RRID: AB_1142428, 1:2000); rabbit anti‐H3K4me1 (Abcam, ab8895, RRID: AB_306847, 1:2500); rabbit anti‐H3K4me3 (Abcam, ab8580, RRID: AB_306649, 1:1000); rabbit anti‐H3K36me2 (Abcam, ab9049, RRID: AB_1280939, 1:1000); rabbit anti‐H3K36me3 (Abcam, ab9050, RRID: AB_306966, 1:1000); rabbit anti‐H3K79me2 (Abcam, ab3594, RRID: AB_303937, 1:2500); rabbit anti‐H3 (Abcam, ab1791, RRID: AB_302613, 1:1000); mouse anti‐Flag (ABclonal, AE005, RRID: AB_2770401, 1:2000); rabbit anti‐HA (Cell Signaling Technology, 11896, RRID: AB_2665471, 1:1000); rabbit anti‐USP10 (Cell Signaling Technology, 8501, RRID: AB_10949976, 1:1000); rabbit anti‐USP13 (Proteintech, 16840‐1‐AP, RRID: AB_2214569, 1:1000); rabbit anti‐USP25 (Proteintech, 12199‐1‐AP, RRID: AB_2212771, 1:500); rabbit anti‐USP28 (Proteintech, 17707‐1‐AP, RRID: AB_2272676, 1:500); rabbit anti‐USP8 (Proteintech, 27791‐1‐AP, RRID: AB_2880973, 1:2000); rabbit anti‐USP2 (Proteintech, 10392‐1‐AP, RRID: AB_2212432, 1:500); rabbit anti‐β‐actin (Proteintech, 66009‐1‐Ig, RRID: AB_2687938, 1:10000); rabbit anti‐CPS1 (Abcam, ab129076, RRID: RRID: AB_11156290, 1:1000); rabbit anti‐ASS1 (Proteintech, 16210‐1‐AP, RRID: AB_2060466, 1:5000); rabbit anti‐ASL (Proteintech, 16645‐1‐AP, RRID: AB_2878293, 1:1000); rabbit anti‐OTC (Proteintech, 26470‐1‐AP, RRID: AB_2880528, 1:500); and rabbit anti‐ARG1 (Proteintech, 16001‐1‐AP, RRID: AB_2289842, 1:5000). Other reagents used in this study included the following: 5MPN (MCE, HY‐123981); monoethyl fumarate (Aladdin, 2459‐05‐4); ATP (Beyotime, D7378); H3B‐120 (MCE, HY‐136128); argininosuccinate (MCE, HY‐113149A); MG132 (Calbiochem, 474790); and cycloheximide (Sigma‐Aldrich, A8244).

### 4.3 Colony Formation Assay

Colony formation assays were performed as previously reported [50, 51]. Specifically, cells in the logarithmic growth phase were digested with trypsin; seeded into six‐well plates at 200, 400, 1000, 3000, and 6000 cells per well; and subjected to 0 Gy, 2 Gy, 4 Gy, 6 Gy, or 8 Gy of irradiation on the second day. The colonies were observed under a microscope after 10–14 days (a cell cluster with more than 50 cells was considered a cell colony). The cell colonies were counted after fixation in methanol and staining with crystal violet. The plating efficiency was determined as the ratio of the colony count per dish to the number of seeded cells. Survival fractions were computed as the plating efficiency of treated cells normalized to the average values of the unirradiated groups [52, 53, 54].

### 4.4 Neutral Comet Assay

Neutral comet assays were performed following the manufacturer's protocol (Trevigen, 4250‐050‐K). Cells were irradiated (6 Gy), incubated for 4 h, and embedded in low‐melting‐point agarose at a 1:10 ratio on comet slides. Following lysis, the slides were subjected to horizontal electrophoresis (21 V, 1 h) in neutral buffer. Then, DNA was precipitated, stained with SYBR Gold, and visualized by fluorescence microscopy. Olive tail moments were quantified using CometScore 2.0 software.

### 4.5 Immunofluorescence Staining

Cells were plated on circular coverslips in 24‐well plates (5 × 10^4^ cells/well) and irradiated (2 Gy) the following day. 4 h post‐irradiation, the cells were fixed, permeabilized, and blocked, followed by incubation with primary antibodies against γH2AX (Abcam, ab81299, 1:800) or Rad51 (Abcam, ab133534, 1:800) at 4°C. After staining with fluorescently labeled secondary antibodies, the foci were visualized by laser confocal microscopy. The foci‐positive cells, defined as those containing > 10 nuclear foci, were quantified.

### 4.6 Construction of PFKFB4‐knockout Cells via CRISPR‐Cas9 Technology

PFKFB4 was ablated using a dual‐sgRNA CRISPR‐Cas9 system targeting sequences flanking exons 8–12 (sgRNA1: 5′‐GTGCGGAGATAGGCCAACAGGGG‐3′; and sgRNA2: 5′‐AGTAGAAAAGCCCCTCATGCAGG‐3′), which resulted in the deletion of the intervening exons. The purity and integrity of the chemically synthesized sgRNAs were validated by gel electrophoresis. Cas9 and sgRNAs were delivered into A549 cells via viral transduction, and single‐cell clones were isolated by limiting dilution in 96‐well plates. PFKFB4 knockout efficiency was confirmed by Western blotting of total protein extracts.

### 4.7 Animal Experiments

Female Balb/c nude mice (4‐5 weeks old) were purchased from Changzhou Cavens Laboratory Animal Co., Ltd. All mice were housed under specific pathogen‐free (SPF) conditions with controlled environmental parameters (temperature: 22 ± 2°C, relative humidity: 50 ± 10%, 12‐h light/dark cycle) and provided with autoclaved food and water ad libitum. All animal experiments were conducted in accordance with the guidelines of the Declaration of Helsinki and the Animals (Scientific Procedures) Act 1986. The sample size for these experiments was determined based on previous studies. Mice were randomly assigned to the experimental groups, and both treatment administration and outcome assessments were performed in a blinded manner.

Xenografts were established by subcutaneous injection of 5 × 10^6^ wild‐type or PFKFB4‐knockdown A549 cells into the right upper flank. When tumors reached an average volume of 100 mm^3^, the mice in the irradiation groups received 10 Gy × 1 irradiation. The tumor dimensions were measured every 3 days, and the tumor volume was calculated as (length × width^2^)/2. The tumors were harvested for examination 15 days after radiation.

5MPN was dissolved in Cremophor EL for in vivo experiments. When the average tumor volume reached 100 mm^3^, the mice received either vehicle or 5MPN (120 mg/kg) via oral gavage every other day according to their assigned groups. The mice in the radiation groups received 10 Gy × 1 irradiation following the initial dose. After the animals were euthanized, the heart, liver, spleen, lung, and kidney tissues were harvested for H&E staining, and peripheral blood was collected to assess the in vivo safety profile of 5MPN.

### 4.8 Immunohistochemical Staining

These experiments were performed on 4 µm sections from paraformaldehyde‐fixed, paraffin‐embedded tissues. After deparaffinization, antigen retrieval, and blocking of endogenous peroxidase (3% H_2_O_2_) and nonspecific sites (5% BSA, 30 min), the sections were incubated with the primary antibody at 4°C overnight. Following washes with PBS, a matched secondary antibody was applied for 1 h at room temperature, and signals were visualized with DAB, followed by counterstaining with hematoxylin and mounting. Staining was evaluated by two pathologists blinded to the groups using a semiquantitative scoring system: IHC score = staining intensity (0, negative; 1, weak; 2, moderate; and 3, strong) × proportion of positive cells (1, < 25%; 2, 26–50%; 3, 51–75%; 4, > 75%). A score ≥ 6 was defined as high expression, and a score < 6 was defined as low expression.

### 4.9 RNA‐seq and RT‐PCR

Total RNA was extracted using a commercial kit (Omega, USA). RNA sequencing was performed by BGI‐Shenzhen. Briefly, poly(A)+ mRNA was enriched using oligo(dT) magnetic beads, fragmented, and used for cDNA synthesis and library construction. Library quality was assessed using a Qubit instrument, and sequencing was conducted on the BGISEQ‐500 platform. Raw sequencing data have been deposited in the Gene Expression Omnibus (GEO) database (https://www.ncbi.nlm.nih.gov/geo/, RRID: SCR_005012) under accession number GSE249850. The transcriptome data were validated by RT‐PCR as previously described [55]. The primer sequences used for RT‐PCR are listed in Table S1.

### 4.10 HM400 High‐throughput Targeted Metabolomics Sequencing

Targeted metabolomic profiling was performed using the HM400 platform (BGI‐Shenzhen). Samples and quality controls (QCs) were homogenized in 50% aqueous methanol, and the supernatants were collected after centrifugation. Serial dilutions of the HM400 standards were used to generate calibration curves. Following derivatization of samples, QCs, and standards, the mixtures were diluted, centrifuged, and analyzed by liquid chromatography‐mass spectrometry (LC‐MS) on a QTRAP 6500+ system (SCIEX). Metabolites were quantified automatically using HMQuant software (BGI) with manual verification. Spearman correlation and variance analyses were conducted using the R packages corr.test and metaX, respectively [56].

### 4.11 Fumarate Assay

Fumarate levels in cell lysates were quantified using a commercial assay kit (Sigma, MAK060). The samples were homogenized in assay buffer and centrifuged at 13,000 × g for 10 min. The supernatant was transferred to a 96‐well plate, mixed with the reaction mixture per the manufacturer's instructions, and incubated for 30 min at room temperature with shaking. The absorbance at 450 nm was measured with an EnSpire 2300 spectrophotometer (USA), and the fumarate concentration was determined from a standard curve.

### 4.12 ^13^C‐Glucose Metabolic Flux Analysis

H1299 cells in the logarithmic growth phase were transiently transfected with either Scramble or siPFKFB4. After 48 h, the medium was replaced with glucose‐free DMEM/F12 containing 10 mmol/L [^1^ ^3^C_6_]‐glucose for isotopic tracing. Cells were harvested 6 h after the medium was changed and processed for LC‐MS analysis following extraction. Water‐soluble metabolites were separated via hydrophilic interaction chromatography (HILIC) using an XBridge BEH Amide column (150 mm × 2.1 mm, 2.5 µm; Waters) and analyzed on a Q Exactive PLUS mass spectrometer (Thermo Scientific). All the data from the isotope labeling experiments were analyzed by El‐MAVEN with MATLAB to correct for natural abundance.

### 4.13 Chromatin Immunoprecipitation (ChIP)

ChIP assay was performed using a commercial kit (Beyotime, P2078) per the manufacturer's protocol. Briefly, DNA and proteins were cross‐linked with formaldehyde, and the chromatin was sheared to 200–800 bp by sonication. Immunoprecipitation was carried out overnight at 4 °C using protein A+G agarose with target‐specific antibodies. After being washed, the complexes were subjected to reverse‐crosslinking by incubation with NaCl at 65 °C for 4 h. The DNA was subsequently purified using a DNA Purification Kit (Beyotime, D0033). Enrichment at the Rad51 promoter was assessed by PCR; the primer sequences are listed in Table S2.

### 4.14 Construction of the Ubiquitinated Compound Library and co‐IP Assay

A549 cells were treated with 198 small molecule inhibitors from a compound library (Selleck, L6000) for 24 h, after which PFKFB4 expression was analyzed by immunoblotting. Candidate compounds that interacted with PFKFB4 were validated by co‐IP. For exogenous co‐IP, S‐protein or myc agarose (Novagen) was added to the cell lysates. For endogenous co‐IP, antibodies against PFKFB4 or USP10 were incubated with lysates in the presence of protein A/G agarose (Santa Cruz Biotechnology). All the mixtures were incubated overnight at 4°C with gentle agitation, followed by five washes with NETN buffer. The beads were then boiled in SDS sample buffer, and protein expression was analyzed by Western blotting.

### 4.15 GST Pull‐Down Assay

The GST‐PFKFB4 plasmid was transformed into *Escherichia coli* for expression. Following bacterial amplification, total protein was extracted by sonication, and the GST‐PFKFB4 fusion protein was subjected to affinity purification. This purified protein was incubated overnight at 4 °C with lysates from HEK293T cells expressing myc‐USP10 together with GST beads (GE Healthcare). After the samples were washed with NETN buffer, the bound proteins were analyzed by Western blotting.

### 4.16 Construction of Domain Deletion Plasmids

On the basis of the literature and molecular structural features [37], defined regions of PFKFB4 and USP10 were deleted. Corresponding primers were designed, and truncated gene fragments were amplified by PCR using the full‐length plasmids as templates. Both the PCR products and the destination vectors were digested with restriction enzymes, and the resulting fragments were subjected to gel purification. These fragments were then ligated into vectors for transformation into competent cells. Positive colonies were isolated and verified by sequencing to confirm that the plasmids were constructed correctly. The primer sequences are listed in Table S3.

### 4.17 In Vivo Ubiquitination Assay

Control and USP10‐silenced lung cancer cells were cotransfected with the SFB‐PFKFB4 and HA‐ubiquitin plasmids for 24 h, followed by treatment with MG132 for 4 h. The cell lysates were incubated with S‐protein agarose (Novagen) overnight at 4°C with gentle agitation. After the cells were washed with NETN buffer, HA‐tagged ubiquitin conjugates were detected by Western blotting to assess PFKFB4 ubiquitination levels.

### 4.18 Mass Spectrometric Identification of the Ubiquitination Sites of PFKFB4

HEK293T cells were transfected with the SFB‐PFKFB4 plasmid for 24 h. Then, 10 µM MG132 was added 6 h before protein extraction. The extracted proteins were enriched with S‐beads and then subjected to mass spectrometry. The modification sites were identified after database searching and filtering of the original mass spectrometry data.

### 4.19 Construction of the Point Mutation Plasmids

SFB‐PFKFB4 point mutations were introduced by site‐directed mutagenesis PCR using KOD Hot Start DNA Polymerase (Sigma‐Aldrich, 71806) and primers designed to incorporate the desired mutation. The PCR products were digested with DpnI to remove the template plasmid and then transformed into JM109 competent cells. Positive clones were selected and verified by sequencing. The sequences of primers used for mutagenesis were as follows.

K431R‐F: GTGGCATATGGTTGTAGAGTGGAGTCCATATTCCTGAACGTGGCT

K431R‐R: GAATATGGACTCCACTCTACAACCATATGCCACAGGAGTCAGCTTCA

### 4.20 Cycloheximide (CHX) Chase Assay

Control and USP10‐silenced lung cancer cells were treated with cycloheximide (CHX; 20 µg/mL) and lysed at the indicated time points. PFKFB4 protein levels were analyzed by Western blotting, with β‐actin serving as the loading control. Band intensities were quantified using ImageJ, and relative protein levels were plotted in GraphPad Prism 10.0 (RRID: SCR_002798) to determine the degradation kinetics and half‐life of PFKFB4.

### 4.21 Statistical Analysis

All in vitro experiments were conducted with at least three biological replicates. Statistically significant differences between two groups were assessed by Student's t test, whereas differences among multiple groups were evaluated using ANOVA. Correlations between categorical variables were analyzed by the chi‐square test. Patient overall survival (OS) was determined by Kaplan‐Meier analysis. Data are presented as the mean ± SD unless otherwise noted. Significance levels are indicated as follows: n.s., *p* > 0.05; *p < 0.05; **p < 0.01; ***p < 0.001.

## Author Contributions

Y.C. and X.J. conceived the study and designed the experiments. Y.C. and Z.W. conducted the experiments. Y.Y. and R.F. analyzed the sequencing data. H.X. contributed to the animal experiments. R.Z. assisted with the study design and discussions during manuscript preparation. G.W. and X.J. provided key intellectual insights and revised the manuscript. All the authors reviewed and approved the final version of the manuscript.

## Ethics Statement

Ethical approval for the human tissue microarray was granted by the Ethics Committee of Shanghai Outdo Biotech Company (SHYJS‐CP‐1701003). All animal experiments were approved by the Medical Ethics Committee of Tongji Medical College, Huazhong University of Science and Technology (IACUC Number: 3606).

## Conflicts of Interest

The authors declare no conflicts of interest.

## Supporting information




**Supporting File 1**: advs76439‐sup‐0001‐SuppMat.docx.

**Supporting File 2**: advs76439‐sup‐0002‐data.zip.

**Supporting File 3**: advs76439‐sup‐0003‐SuppMat.docx.

## Data Availability

The data that support the findings of this study are available from the corresponding author upon reasonable request.
