# Supplementary material for: PFKFB4 Deubiquitination by USP10 Enhances Fumarate Metabolism to Orchestrate the KDM1A/Rad51 Axis and Confer Radioresistance in Lung Cancer
Source: Adv Sci (Weinh). 2026 Jul 8;13(54):e76439. doi: 10.1002/advs.76439 (PMC13344063; doi:10.1002/advs.76439)
Supplement: Supplementary file 1 [file ADVS-13-e76439-s001.docx]

**Supporting Information-Supplemental Figures and Tables**

**PFKFB4 Deubiquitination by USP10 Enhances Fumarate Metabolism to Orchestrate the KDM1A/Rad51 Axis and Confer Radioresistance in Lung Cancer**

Yunshang Chen^1,2,3^ | Zilong Wu^1,2,3^ | Yongqiang Yang^1,2,3^ | Ruoxin Fang^1,2,3^ | Huichan Xue^1,2,3^ | Rui Zhou^1,2,3^ | Gang Wu^1,2,3^ | Xiaohua Jie^1,2,3^

^1^Cancer Center, Union Hospital, Tongji Medical College, Huazhong University of Science and Technology, Wuhan 430022, China | ^2^Institute of Radiation Oncology, Union Hospital, Tongji Medical College, Huazhong University of Science and Technology, Wuhan 430022, China | ^3^Hubei Key Laboratory of Precision Radiation Oncology, Wuhan 430022, China

**Correspondence:** Rui Zhou (mimiruirui2@163.com) | Gang Wu (xhzlwg@163.com) | Xiaohua Jie (xhzljxh@hust.edu.cn)

**Keywords:** Non-small cell lung cancer | Radioresistance | PFKFB4 | Fumarate | Deubiquitination

Yunshang Chen and Zilong Wu contributed equally to this work.

**FIGURE S1** | PFKFB4 is highly expressed in lung cancer cells and is associated with poor outcomes in patients treated with radiotherapy. (A) PFKFB4 expression is higher in lung adenocarcinoma tissues than in normal tissues (data from UALCAN). (B) PFKFB4 expression is elevated in lung cancer cell lines (A549, H1299, H827, and H460) compared with that in the normal human bronchial epithelial cell line HBE. (C) Bar graph showing the relative expression levels of PFKFB4 in different cell lines. n = 3. (D) IHC staining for PFKFB4 was performed in lung adenocarcinoma tissue microarrays, and representative images are presented. Scale bar: 50 µm. (E) The expression level of PFKFB4 was significantly greater in lung cancer tissues than in paracancerous tissues (n = 80). (F) Survival curves for lung cancer patients with high and low PFKFB4 expression. Significance is shown in the figure: ****p* < 0.001.

**FIGURE S2** | PFKFB4 protein levels are decreased by siRNAs and sgRNAs. (A) A549 and H1299 cells were transfected with the corresponding siRNAs, and proteins were harvested after 48 h and subjected to Western blotting. (B) Western blotting confirmed the successful construction of the PFKFB4-knockdown A549 cell line.

**FIGURE S3** | The IC50 of 5MPN was determined by CCK8 assays. (A,B) 5MPN was dissolved in DMSO and serially diluted for cell treatment, with the final DMSO concentration fixed at 0.1%. Following a 24-hour incubation, cell viability was measured by CCK-8 assay. Based on the results, 100 μM 5MPN was chosen for subsequent experiments.

**FIGURE S4** | Representative images of colony formation with targeted inhibition of PFKFB4. (A) Representative images of colony formation in the control and PFKFB4-silenced groups after irradiation at various doses. (B) Representative images of colony formation in the groups treated with DMSO and 5MPN after irradiation at various doses.

**FIGURE S5** | 5MPN does not affect the radiosensitivity of HBE cells. (A) Survival curves of HBE cells treated with DMSO or 5MPN. n = 3. (B) Representative images of colonies formed by HBE cells treated with DMSO or 5MPN. Significance is shown in the figure: n.s., *p* > 0.05.

**FIGURE S6** | Targeted inhibition of PFKFB4 synergizes with radiotherapy in vivo*.* (A) Representative images of Ki67 IHC staining of subcutaneous tumors and statistical analysis of the IHC scores. n = 5. Scale bar: 50 µm. (B) Photographs of subcutaneous tumors excised from the indicated groups of mice. n = 6. (C) Representative images of Ki67 IHC staining in subcutaneous tumors are presented, followed by statistical analysis of the corresponding IHC scores. n = 5. Scale bar: 50 µm. Significance is shown in the figure: **p* < 0.05; ***p* < 0.01; ****p* < 0.001.

**FIGURE S7** | Knockdown of PFKFB4 induces alterations in the gene expression profile. (A) Transcriptomic analysis upon PFKFB4 knockdown using two independent siRNAs. The number of DEGs is shown (Q < 0.05, |fold change| ≥ 2). (B) Venn diagram of DEGs after treatment with siPFKFB4 #1 and #2 compared with the control.

**FIGURE S8** | Targeted inhibition of PFKFB4 downregulates Rad51 expression. (A,B) Cells were irradiated (2 Gy) and subjected to Rad51 immunofluorescence staining 4 h later. Representative images and statistical analysis of foci-positive cells are shown. n = 3. Scale bar: 10 µm. (C) IHC staining images of Rad51 and PFKFB4 in subcutaneous tumors, with representative images and scoring statistics. n = 5. Scale bar: 50 µm. Significance is shown in the figure: ****p* < 0.001.

**FIGURE S9** | Silencing of PFKFB4 induces distinct global metabolic profiles. (A) Principal component analysis (PCA) of the targeted metabolomics sequencing data. (B) Partial least squares-discriminant analysis (PLS-DA) of the targeted metabolomics sequencing data.

**FIGURE S10** | Targeted inhibition of PFKFB4 does not alter fumarate levels in HBE cells. (A) Intracellular fumarate levels (nmol/10^6^ cells) in control and PFKFB4-silenced HBE cells. n = 3. (B) Intracellular fumarate levels (nmol/10^6^ cells) in HBE cells upon treatment with DMSO or 5MPN. n = 3. Significance is shown in the figure: n.s., *p* > 0.05.

**FIGURE S11** | The expression of key urea cycle enzymes is independent of PFKFB4 regulation. Protein expression in A549 cells was assessed by Western blotting 48 h after transfection with scramble or siPFKFB4.

**FIGURE S12** | KDM4A, KDM4C, and KDM5B are not involved in the fumarate-mediated regulation of Rad51. Western blotting of Rad51 expression in A549 cells transfected with the respective plasmids and subsequently treated with fumarate.

**FIGURE S13** | Schematic of the ubiquitinated compound library construction process.

**FIGURE S14** | Analysis of the GEPIA database revealed the conserved role of the USP10/PFKFB4 regulatory axis across additional cancer types. (A-D) High PFKFB4 expression is associated with poor patient outcomes. (E-H) USP10 expression is positively correlated with PFKFB4 levels. Abbreviations: CESC: cervical squamous cell carcinoma; KIRP: kidney renal papillary cell carcinoma; LIHC: liver hepatocellular carcinoma; MESO: mesothelioma.

| **Genes** | **Sequences (5'--3')** |
| --- | --- |
| PFKFB4 | F: CACAAACACCACCCGAGAAC |
|  | R: TCCTCCGTAGCCTCATCACT |
| Rad51 | F: GCTGATGAGTTTGGTGTAG |
|  | R: TTTTGCAGATTCTGGTTT |
| RAD54L | F: CGCCAGAGTCCAGAGTGC |
|  | R: TCCGAGCCATTTCCCAAC |
| XRCC2 | F: TTTATCACCTAACAGCACG |
|  | R: TCTTCCCAGGCAGTATTT |
| POLD2 | F: TGGCTCCAAAATCATCCG |
|  | R: GCCTCCCAGGTCATCGTC |
| BRCA1 | F: ATAATACAAGAGCGTCCCC |
|  | R: CCGTTTGGTTAGTTCCCT |

F, forward primer; R, reverse primer.

**TABLE S1** | Sequences of primers used for RT-PCR.

| Genes | Primer | **Sequences (5'--3')** |
| --- | --- | --- |
| Rad51 | Primer 1 | F: AAACAGAAGACGGCAACTCG |
|  |  | R: CGCGAGTTTACAGACTGCC |
|  | Primer 2 | F: AAGCACCACAGATTGACGAA |
|  |  | R: TTGTGGTTTGTTTCGGCAGT |
|  | Primer 3 | F: CCTTCCAGTTTCGGCACTTG |
|  |  | R: TTGCAGCCAACCGAGATCT |

F, forward primer; R, reverse primer.

**TABLE S2** | Sequences of primers used for ChIP-PCR.

| Genes | Domain | **Sequences (5'--3')** |
| --- | --- | --- |
| PFKFB4 | aa 1--249 | F: ATCCACGTGACCCCCGGATCCAAAGAAACC  GCTGCTGCTA |
|  |  | R: GGTTTCTTTGGATCCGGGGGTCACGTGGAT  GTTCATGAG |
|  | aa 250--469 | F: AAGCTGGCTAGCCACCATGCGCTCCATCTA  CCTCTGCCGGCA |
|  |  | R: GAGGTAGATGGAGCGCATGGTGGCTAGCC  AGCTTGGGTCTC |
| USP10 | aa 1--100 | F: CTTGGTACCGAGCTCGGATCCGCCACCATG  GCCCTCCACAG |
|  |  | R: TATTAGTTTTTGTTCCTCGAGGGTTATTTTGG  AAGCTGTACAACCGAGAATAAATTCA |
|  | aa 101--399 | F: CTTGGTACCGAGCTCGGATCCGCCACCATGC  CTGATGGTATCACTAAAGAAGC |
|  |  | R: TATTAGTTTTTGTTCCTCGAGCAACTCTGCA  ATCTTTATGGC |
|  | aa 400--798 | F: ACCGAGCTCGGATCCGCCACCATGCTGGAG  AATGTAACCCTAATCCATAAACC |
|  |  | R: ATATTAGTTTTTGTTCCTCGAGCAGCAGGTC  CACTCGGCGGTA |

F, forward primer; R, reverse primer.

**TABLE S3** | Sequences of primers used for constructing domain deletion plasmids of PFKFB4 and USP10.
