# Supplementary material for: PFKFB4 Deubiquitination by USP10 Enhances Fumarate Metabolism to Orchestrate the KDM1A/Rad51 Axis and Confer Radioresistance in Lung Cancer
Source: Adv Sci (Weinh). 2026 Jul 8;13(54):e76439. doi: 10.1002/advs.76439 (PMC13344063; doi:10.1002/advs.76439)
Supplement: Supplementary file 2 [file ADVS-13-e76439-s002.docx]

**Supporting Information-Full unedited western blots**

**PFKFB4 Deubiquitination by USP10 Enhances Fumarate Metabolism to Orchestrate the KDM1A/Rad51 Axis and Confer Radioresistance in Lung Cancer**

Yunshang Chen^1,2,3^ | Zilong Wu^1,2,3^ | Yongqiang Yang^1,2,3^ | Ruoxin Fang^1,2,3^ | Huichan Xue^1,2,3^ | Rui Zhou^1,2,3^ | Gang Wu^1,2,3^ | Xiaohua Jie^1,2,3^

^1^Cancer Center, Union Hospital, Tongji Medical College, Huazhong University of Science and Technology, Wuhan 430022, China | ^2^Institute of Radiation Oncology, Union Hospital, Tongji Medical College, Huazhong University of Science and Technology, Wuhan 430022, China | ^3^Hubei Key Laboratory of Precision Radiation Oncology, Wuhan 430022, China

**Correspondence:** Rui Zhou (mimiruirui2@163.com) | Gang Wu (xhzlwg@163.com) | Xiaohua Jie (xhzljxh@hust.edu.cn)

**Keywords:** Non-small cell lung cancer | Radioresistance | PFKFB4 | Fumarate | Deubiquitination

Yunshang Chen and Zilong Wu contributed equally to this work.

**Full unedited Western blots** | Black boxes indicate the images used in Figure 2 and Figure 4.

**Full unedited Western blots** | Black boxes indicate the images used in Figure 5.

**Full unedited Western blots** | Black boxes indicate the images used in Figure 6.

**Full unedited Western blots** | Black boxes indicate the images used in Figure 7 and Figure S1.

**Full unedited Western blots** | Black boxes indicate the images used in Figure S2, Figure S11, and Figure S12.
